# Construction of Space-Filling Asymmetrical Marginally Coupled Designs

**DOI:** 10.3390/e27121256

**Published:** 2025-12-13

**Authors:** Weiping Zhou, Miaomiao Meng, Min Li, Xue Yang

**Affiliations:** 1 School of Mathematics and Computing Science, Guangxi Colleges and Universities Key Laboratory of Data Analysis and Computation, Guilin University of Electronic Technology, Guilin 541004, China; wpzhou@guet.edu.cn (W.Z.); mmyam@mails.guet.edu.cn (M.M.); 2 Center for Applied Mathematics of Guangxi (GUET), Guilin 541004, China; 3 Guangxi Academy of Artificial Intelligence, Nanning 530028, China; 4 School of Mathematics and Statistics, Qingdao University, Qingdao 266071, China; 5 School of Statistics, Tianjin University of Finance and Economics, Tianjin 300222, China

**Keywords:** computer experiment, projection, space-filling property, stratification, 62K05, 62K99

## Abstract

Marginally coupled designs (MCDs) are very suitable for computer experiments with both qualitative and quantitative factors. An MCD consisting of two subdesigns—one for the qualitative factors and the other for the quantitative factors—is said to be symmetrical or asymmetrical when the qualitative factor subdesign is equal-level or mixed-level, respectively. Although symmetrical MCDs have been studied extensively recently, investigations of asymmetrical MCDs are still relatively scarce. In this paper, based on space-filling symmetrical MCDs or space-filling Latin hypercube designs (LHDs), we propose four approaches to constructing a series of space-filling asymmetrical MCDs. The obtained asymmetrical MCDs can inherit the low-dimensional space-filling properties of these symmetrical MCDs or these LHDs. Moreover, the resulting asymmetrical MCDs are flexible in terms of their run sizes. A numerical study is conducted to compare and evaluate the performance of the proposed designs in computer experiments.

## 1. Introduction

Space-filling designs are extremely appropriate for computer experiments that are employed to address complex scientific or engineering problems. Using such designs to plan experiments can spread points in the experimental domain as evenly as possible, which allows every region of the experimental domain to be explored [1,2]. Hence, space-filling designs help to build predictions with high average accuracy when making predictions for unsampled points [3]. Some researchers, such as [4,5], have worked on the construction of space-filling designs using Monte Carlo methods; some scholars have proposed obtaining such designs using entropy criterion optimization (see [3,6,7,8,9,10]); others have constructed such designs based on space-filling properties, such as the maximin distance criterion, discrepancy criterion, and low-dimensional projection [3]. Maximum entropy designs can be approximated as maximin distance designs under the assumption of very weak correlations [3,11,12]. The common goal of Monte Carlo methods, entropy optimization, and space-filling properties is to maximize the information obtained from complex systems using economical run sizes in experiments. Consequently, space-filling designs can be viewed as the interdisciplinary intersection of statistics and information theory. In this paper, we investigate a class of space-filling designs suitable for computer experiments with both qualitative and quantitative factors.

Computer experiments with both qualitative and quantitative factors represent a considerably effective methodology for investigating complex systems and addressing scientific challenges [13,14,15]. Extensive studies have been conducted on how to efficiently plan such experiments. This goes back to Qian et al. [16] and subsequently Qian [17], who considered systematically planning such experiments using sliced space-filling designs and sliced Latin hypercube designs (SLHDs), respectively. But, these two types of designs can be criticized for their cost–benefit restrictions, namely that the run sizes increase dramatically as the number of qualitative factors grows. For cost-effectiveness, marginally coupled designs (MCDs), with economical run sizes and accommodating a large number of qualitative factors, are proposed by Deng et al. [18]. An MCD consists of two subdesigns, one of which is used to arrange qualitative factors and the other to arrange quantitative factors. In an MCD, the qualitative factor subdesign is commonly an orthogonal array (OA) [19], the quantitative factor subdesign is a Latin hypercube design (LHD) [20], and the latter subdesign is also an SLHD with respect to any qualitative factor in the former subdesign. Since MCDs were proposed, scholars have worked to improve them to have superior properties. Some researchers have worked on optimizing properties of the subdesign for quantitative factors in an MCD, such as improving the low-dimensional space-filling property [21,22,23,24] and orthogonality [25]. Others have concentrated on enhancing the stratification property between any *l* (l≤2) qualitative factors and all quantitative factors, such as doubly coupled designs (DCDs) [26], group doubly coupled designs (GDCDs) [27], and strongly coupled designs (SCDs) [28]. A design with both qualitative and quantitative factors is classified as symmetrical if the subdesign of qualitative factors is a fixed-level OA. Conversely, such a design is termed asymmetrical when the subdesign of the qualitative factors employs a mixed-level OA. The above MCDs, DCDs, GDCDs, and SCDs are predominantly symmetrical, while asymmetrical designs are relatively rare.

In this paper, we propose four approaches to constructing asymmetrical MCDs based on symmetrical MCDs. When the symmetrical MCDs with low-dimensional space-filling properties in subdesigns of quantitative factors exist, the subdesigns of quantitative factors in the asymmetrical MCDs obtained by Algorithms 1 and 4 can inherit these space-filling properties. The space-filling symmetrical MCDs can be obtained from He et al. [21,22,23] and Yang et al. [26]. If LHDs with desirable low-dimensional space-filling properties are available, Algorithms 2 and 3 can be employed to create asymmetrical MCDs in which the subdesigns of quantitative factors have the same space-filling properties as those of the LHDs. Orthogonal-array-based LHDs and strong-orthogonal-array-based LHDs have been thoroughly examined in the existing literature [29,30,31,32,33,34,35]; therefore, Algorithms 2 and 3 can employ these types of LHDs. Moreover, the obtained asymmetrical MCDs are flexible in terms of their run sizes.

The remainder of the paper is organized as follows. Section 2 gives some definitions and notation. Section 3 proposes several construction methods of asymmetrical MCDs. In Section 4 we conduct a numerical study to verify the performance of the proposed designs. Concluding remarks are provided in Section 5. All proofs and some tables are provided in Appendix A and Appendix B, respectively.

## 2. Definitions and Notation

Throughout this paper, $0_{n}$ is an n×1 vector of zeros, $1_{n}$ is an n×1 vector of ones, and $J_{u\times q}$ is a u×q matrix of ones. For two matrices, $A={\left(a_{ij}\right)}_{n\times m}$ and $B={\left(b_{ij}\right)}_{p\times q}$, their Kronecker sum and Kronecker product are defined as $A⊕B={\left(a_{ij}+B\right)}_{\left(np)\times (mq\right)}$ and $A⊗B={\left(a_{ij}\times B\right)}_{\left(np)\times (mq\right)}$, respectively. Let $D={\left(d_{ij}\right)}_{n\times m}$ be an n×m matrix, and for a given integer *s*, s≥2, define an n×m matrix ψ(D,s) as

(1) $$\psi \left(D,s\right)={\left(\lfloor d_{ij}/s\rfloor \right)}_{n\times m},$$

where $\left\lfloor x\right\rfloor$ represents the largest integer not exceeding *x*.

Let GF(s) denote the Galois field of order *s*, with each of the *s* levels coming from $\left\{\alpha_{0},\alpha_{1},\ldots ,\alpha_{s-1}\right\}$, where $\alpha_{0}=0$ and $\alpha_{1}=1$. If *s* is a prime, $GF\left(s\right)=\left\{\alpha_{0},\alpha_{1},\ldots ,\alpha_{s-1}\right\}$ simplifies to $GF\left(s\right)=\left\{0,1,\ldots ,s-1\right\}$. A difference scheme of strength 2 with *u* rows, *v* columns, and *s* levels, denoted by D(u,v,s), is an u×v matrix with entries taken from GF(s) such that in the vector difference between any two distinct columns, the *s* levels in GF(s) occur with the same frequency. An n×q matrix is called an orthogonal array (OA) of strength *t*, denoted as $OA\left(n,s_{1}^{q_{1}}s_{2}^{q_{2}}\cdots s_{v}^{q_{v}},t\right)$, $q_{1}+q_{2}+\cdots +q_{v}=q$; if (i) the entries of the first $q_{1}$ columns are taken from $\left\{0,1,2,\ldots ,s_{1}-1\right\}$, the entries of the next $q_{2}$ columns are taken from $\left\{0,1,2,\ldots ,s_{2}-1\right\}$, and so on; (ii) all possible *t*-tuples occur equally often in any *t* columns. The $OA\left(n,s_{1}^{q_{1}}s_{2}^{q_{2}}\cdots s_{v}^{q_{v}},t\right)$ with $s_{1}=s_{2}=\cdots =s_{v}=s$ is called a fixed-level or symmetrical OA and denoted by $OA\left(n,s^{q},t\right)$; otherwise, the array is called a mixed-level or asymmetrical OA. An n×p matrix is called a Latin hypercube design (LHD) with *n* runs and *p* factors, denoted as $LHD\left(n,p\right)$, if each column of the matrix contains *n* levels taken from $\left\{0,1,2,\ldots ,n-1\right\}$ [20]. Let $D={\left(d_{ij}\right)}_{n\times m}$ be a matrix of *n* runs, *m* factors, and *w* levels, and for 2≤t≤m, the *t* columns of *D* are said to achieve *t*-dimensional stratification on an $s_{1}\times \cdots \times s_{t}$ grid if the *t* columns can be collapsed into an $OA\left(n,s_{1}s_{2}\cdots s_{t},t\right)$, where the *w* levels are collapsed into $s_{k}$ levels by $\psi (d_{ij},w/s_{k})$ in Equation (1).

A design $D=(D_{1},D_{2})$, with $D_{1}$ and $D_{2}$ representing two subdesigns for qualitative and quantitative factors, respectively, is called a marginally coupled design (MCD) if (i) $D_{1}$ and $D_{2}$ are an OA and an LHD, respectively, and (ii) the rows in $D_{2}$ corresponding to each level of any factor in $D_{1}$ form a small LHD. If $D_{1}$ and $D_{2}$ are an $OA\left(n,s^{q},t\right)$ and an $LHD\left(n,p\right)$, respectively, the MCD is symmetric and denoted by $MCD\left(n,s^{q},p\right)$; if $D_{1}$ and $D_{2}$ are an $OA\left(n,s_{1}^{q_{1}}s_{2}^{q_{2}}\cdots s_{v}^{q_{v}},t\right)$ and an $LHD\left(n,p\right)$, respectively, the MCD is asymmetric and denoted by $MCD\left(n,s_{1}^{q_{1}}s_{2}^{q_{2}}\cdots s_{v}^{q_{v}},p\right)$.

The necessary and sufficient condition for the existence of a symmetrical MCD is presented by Proposition 1 of He et al. [21]. Building upon their work, we can directly derive a necessary and sufficient condition for the existence of an asymmetrical MCD as follows.

**Lemma 1.** 
*Suppose $D_{1}=\left(D_{11},D_{12},\ldots ,D_{1m}\right)$ is an $OA\left(n,s_{1}^{q_{1}}s_{2}^{q_{2}}\cdots s_{m}^{q_{m}},2\right)$, where $D_{1i}$ is an $OA\left(n,s_{i}^{q_{i}},2\right)$ for i=1,2,…,m, and $D_{2}$ is an $LHD\left(n,p\right)$; then $D=\left(D_{1},D_{2}\right)$ is an $MCD\left(n,s_{1}^{q_{1}}s_{2}^{q_{2}}\cdots s_{m}^{q_{m}},p\right)$ if and only if $\left(D_{1i},\psi \left(d_{2},s_{i}\right)\right)$ is an $OA\left(n,s_{i}^{q_{i}}\left(n/s_{i}\right),2\right)$, where $d_{2}$ is any column of $D_{2}$, and $\psi (d_{2},s_{i})$ is obtained from Equation (1), i=1,2,…,m.*

Note that, When m=1, Lemma 1 is transformed into Proposition 1 of He et al. [21].

## 3. Construction of Asymmetrical MCDs with Low-Dimensional Stratification

This section introduces four construction methods that utilize symmetrical MCDs to construct asymmetrical MCDs. Furthermore, the low-dimensional projection properties of the proposed MCDs are also investigated.

The first method presents a construction for an $MCD\left(2n,2^{m-1}4^{1},p\right)$, $\left(D_{1},D_{2}\right)$, through an $MCD\left(n,2^{m},p\right)$, $\left(A_{1},A_{2}\right)$. The key feature of $\left(D_{1},D_{2}\right)$ is that the space-filling property of $D_{2}$ is determined by that of $A_{2}$. Suppose an $MCD\left(n,2^{m},p\right)$, denoted as $A=(A_{1},A_{2})$, is available, where $A_{1}$ and $A_{2}$ are an $OA\left(n,2^{m},2\right)$ and an $LHD\left(n,p\right)$, respectively. For clarity, let the Kronecker sum ⊕ in Algorithm 1 be defined over GF(2). Algorithm 1 is as follows.
**Algorithm 1** Construction of $MCD\left(2n,2^{m-1}4^{1},p\right)$ based on $MCD\left(n,2^{m},p\right)$*Step* *1.*Let $a_{11}^{0}={\left(0,1\right)}^{T}⊗1_{n}$ and $a_{11}^{i}={\left(0,1\right)}^{T}⊕a_{1}^{i}$ over GF(2), where $a_{1}^{i}$ is the *i*th column of $A_{1}$ for i=1,2,…,m. Obtain a (2n)×m matrix $D_{1}=\left(a_{11}^{1},a_{11}^{2},\ldots ,a_{11}^{m-1},2a_{11}^{m}+a_{11}^{0}\right)$.*Step* *2.*Construct a 2n×p matrix ${\tilde{D}}_{2}=1_{2}⊗A_{2}$. Obtain a 2n×p matrix $D_{2}$ based on ${\tilde{D}}_{2}$ by replacing the 2 entries with level *k* in each column of ${\tilde{D}}_{2}$ by a random permutation of {2k,2(k+1)−1} for k=0,1,…,n−1.*Step* *3.*The resulting design $D=(D_{1},D_{2})$.

**Theorem** **1.** *For $A=(A_{1},A_{2})$ in Algorithm 1 and $D=(D_{1},D_{2})$ generated by Algorithm 1, the following are true:*

(i) *$D=(D_{1},D_{2})$ is an $MCD\left(2n,2^{m-1}4^{1},p\right)$;*
(ii) *$D_{2}$ and $A_{2}$ achieve the same low-dimensional stratification.*

Obviously, (i) $A=(A_{1},A_{2})$ and $D=(D_{1},D_{2})$ are symmetrical and asymmetrical MCDs, respectively, and (ii) if $A_{2}$ achieves stratification in any *t* dimensions, $D_{2}$ also achieves the same *t*-dimensional stratification as $A_{2}$.

**Remark 1.** 
*The $MCD\left(n,2^{m},p\right)$, $A=(A_{1},A_{2})$, in Algorithm 1 can be obtained from He et al. [21]. For a given integer λ, λ≥1, an $MCD\left(8\lambda ,2^{4\lambda },4\lambda -1\right)$ can be generated by Construction 1 of He et al. [21]; then an $MCD\left(16\lambda ,2^{4\lambda -1}4^{1},4\lambda -1\right)$ $\left(D_{1},D_{2}\right)$ exists and $D_{2}$ achieves stratification on 2×2 grids. For a given integer u, u≥3, a space-filling $MCD\left(2^{u},2^{m},2\right)$ with $m=2^{u-2}$ can be obtained through Construction 2 of He et al. [21]; then an $MCD\left(2^{u+1},2^{m-1}4^{1},2\right)$ $\left(D_{1},D_{2}\right)$ exists, and $D_{2}$ achieves stratification on $2^{u-1}\times 2$ and $2\times 2^{u-1}$ grids. For a given integer u, u≥2, k=1,2, $m=\left(3-k\right)2^{u-2}$, and p=k(u−1), a space-filling $MCD\left(2^{u},2^{m},p\right)$ can be obtained via Construction 3 of He et al. [21]; then (i) an $MCD\left(2^{u+1},2^{m-1}4^{1},p\right)$ $\left(D_{1},D_{2}\right)$ exists, and (ii) $D_{2}$ can be partitioned into k disjoint groups of u−1 columns; any two distinct columns in $D_{2}$ achieve stratification on a 2×2 grid; any two columns from different groups in $D_{2}$ achieve stratification on $2^{u-1}\times 2$ and $2\times 2^{u-1}$ grids. Moreover, $A=(A_{1},A_{2})$ in Algorithm 1 can also be taken from He et al. [23], and similar results can be obtained.*

**Example 1.** 
*Consider n=8, m=4, and p=3, and let $A=(A_{1},A_{2})$ be an $MCD\left(8,2^{4},3\right)$, which are obtained from Construction 1 of He et al. [21] and listed as follows:*

$$A_{1}={\left(\begin{array}{cccccccc} 0 & 1 & 0 & 1 & 0 & 1 & 0 & 1\\ 0 & 1 & 0 & 1 & 1 & 0 & 1 & 0\\ 0 & 1 & 1 & 0 & 0 & 1 & 1 & 0\\ 0 & 1 & 1 & 0 & 1 & 0 & 0 & 1 \end{array}\right)}^{T}and\;\;A_{2}={\left(\begin{array}{cccccccc} 5 & 4 & 6 & 7 & 0 & 1 & 2 & 3\\ 7 & 6 & 2 & 3 & 4 & 5 & 1 & 0\\ 2 & 3 & 4 & 5 & 6 & 7 & 1 & 0 \end{array}\right)}^{T}.$$

*Let $a_{1}^{i}$ be the ith column of $A_{1}$ for i=1,2,3,4, i.e., $A_{1}=\left(a_{1}^{1},a_{1}^{2},a_{1}^{3},a_{1}^{4}\right)$. Then $a_{11}^{i}$ for i=0,1,…,4 and $D_{1}$ are found to be $a_{11}^{0}={\left(0,1\right)}^{T}⊗1_{8}$, $a_{11}^{1}={\left(0,1\right)}^{T}⊕a_{1}^{1}$, $a_{11}^{2}={\left(0,1\right)}^{T}⊕a_{1}^{2}$, $a_{11}^{3}={\left(0,1\right)}^{T}⊕a_{1}^{3}$, $a_{11}^{4}={\left(0,1\right)}^{T}⊕a_{1}^{4}$, and $D_{1}=\left(a_{11}^{1},a_{11}^{2},a_{11}^{3},2a_{11}^{4}+a_{11}^{0}\right)$, listed as follows:*

$$D_{1}={\left(\begin{array}{cccccccccccccccc} 0 & 1 & 0 & 1 & 0 & 1 & 0 & 1 & 1 & 0 & 1 & 0 & 1 & 0 & 1 & 0\\ 0 & 1 & 0 & 1 & 1 & 0 & 1 & 0 & 1 & 0 & 1 & 0 & 0 & 1 & 0 & 1\\ 0 & 1 & 1 & 0 & 0 & 1 & 1 & 0 & 1 & 0 & 0 & 1 & 1 & 0 & 0 & 1\\ 0 & 2 & 2 & 0 & 2 & 0 & 0 & 2 & 3 & 1 & 1 & 3 & 1 & 3 & 3 & 1 \end{array}\right)}^{T}.$$

*Construct a 16×3 matrix ${\tilde{D}}_{2}$ where ${\tilde{D}}_{2}=1_{2}⊗A_{2}$; then we can obtain $D_{2}$ as follows:*

$$D_{2}={\left(\begin{array}{cccccccccccccccc} 10 & 8 & 12 & 14 & 0 & 2 & 4 & 6 & 11 & 9 & 13 & 15 & 1 & 3 & 5 & 7\\ 14 & 12 & 4 & 6 & 8 & 10 & 2 & 0 & 15 & 13 & 5 & 7 & 9 & 11 & 3 & 1\\ 4 & 6 & 8 & 10 & 12 & 14 & 2 & 0 & 5 & 7 & 9 & 11 & 13 & 15 & 3 & 1 \end{array}\right)}^{T}.$$

**Figure 1:**
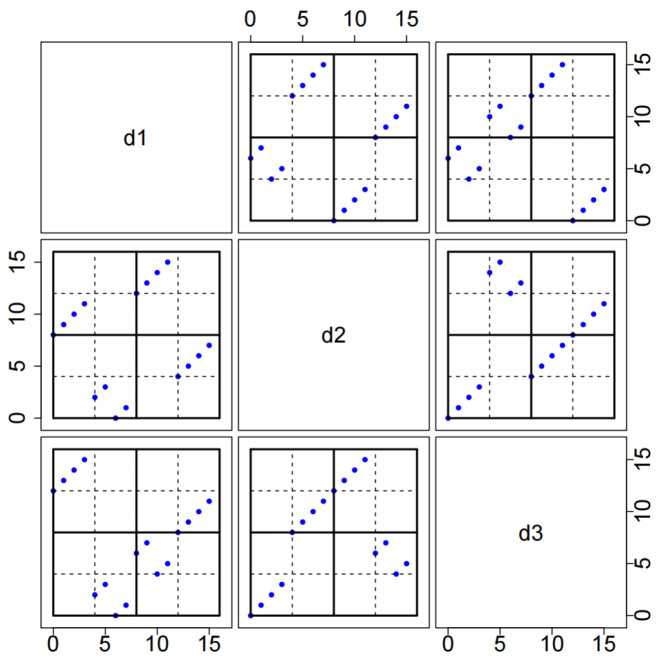
Bivariate projections among the three columns $d_{1}$, $d_{2}$, $d_{3}$ of $D_{2}$ in Example 1.

*It is easy to check that $\left(D_{1},D_{2}\right)$ is an $MCD\left(16,2^{3}4^{1},3\right)$. The two-dimensional space-filing properties of $D_{2}$ can be seen intuitively in Figure 1, i.e., any two distinct columns in $D_{2}$ achieve stratification on a 2×2 grid.*

Algorithm 1 provides a way to construct $MCD\left(2n,2^{m-1}4^{1},p\right)$s with low-dimensional stratification via $MCD\left(n,2^{m},p\right)$s with good properties in low-dimensional projections. In the following, we present an approach to constructing MCDs with $u_{1}n$ runs with $u_{1}=\lambda s$ and λ≥1. Suppose a difference scheme $D(u_{1},v_{1},s)$, denoted as $\Lambda_{1}$, and an $MCD\left(n,s^{m},p\right)$, denoted as $B=(B_{1},B_{2})$, are available, where $B_{1}$ and $B_{2}$ are an $OA\left(n,s^{m},2\right)$ and an $LHD\left(n,p\right)$, respectively. Algorithm 2 offers a method for constructing asymmetrical MCDs, as detailed below.
**Algorithm 2** Construction of $MCD\left(nu_{1},s^{mv_{1}^{*}}n^{1},qp\right)$ based on $MCD\left(n,s^{m},p\right)$*Step 1.* 
Let $\Lambda_{1}^{*}=\Lambda_{1}\setminus 0_{u_{1}}$, where $\Lambda_{1}\setminus 0_{u_{1}}$ is the matrix that consists of all columns in $\Lambda_{1}$ except $0_{u_{1}}$. Let $v_{1}^{*}=v_{1}-1$ if $0_{u_{1}}$ is in $\Lambda_{1}$; otherwise, let $v_{1}^{*}=v_{1}$.*Step 2.* 
Construct an $\left(nu_{1}\right)\times \left(mv_{1}^{*}+1\right)$ matrix as $D_{1}=\left(\Lambda_{1}^{*}⊕B_{1},1_{u_{1}}⊗{\left(0,1,\ldots ,n-1\right)}^{T}\right)$, where the ⊕ operator is based on GF(s).*Step 3.* 
For a given q≥1, let $D_{2}=J_{u_{1}\times q}⊗B_{2}+n\Pi_{1}⊗1_{n}$, where $\Pi_{1}$ is an $LHD\left(u_{1},qp\right)$.*Step 4.* 
The resulting design $D=(D_{1},D_{2})$.

**Theorem** **2.** *For $\Pi_{1}$ from Step 3 of Algorithm 2 and $D=(D_{1},D_{2})$ generated by Algorithm 2, the following are true:*

(i) *$D=(D_{1},D_{2})$ is an $MCD\left(nu_{1},s^{mv_{1}^{*}}n^{1},qp\right)$, if $0_{u_{1}}$ is in $\Lambda_{1}$, $v_{1}^{*}=v_{1}-1$; otherwise $v_{1}^{*}=v_{1}$;*
(ii) *$D_{2}$ and $\Pi_{1}$ achieve the same low-dimensional stratification.*

The space-filling property of $\Pi_{1}$ plays a critical role in the space-filling property of $D_{2}$ in Theorem 2, since $\psi \left(D_{2},n\right)=\Pi_{1}⊗1_{n}$. More precisely, if the LHD $\Pi_{1}$ is based on an OA of strength *t*, $D_{2}$ will have stratification in any *t* dimensions; if it is based on a strong orthogonal array $SOA(u_{1},qp,\theta^{3},3)$ [31], $D_{2}$ will achieve stratification on the θ×θ×θ grids in any three dimensions; in addition, it can achieve stratification on the $\theta^{2}\times \theta$ and the $\theta \times \theta^{2}$ grids in any two dimensions. The LHD $\Pi_{1}$ can be obtained from orthogonal-array-based LHDs or strong-orthogonal-array-based LHDs [29,30,31,32,33,34,35].

**Example 2.** 
*Let $\Lambda_{1}$ be a $D\left(8,8,2\right)$, $B=(B_{1},B_{2})$ be an $MCD\left(4,2^{2},1\right)$, and $\Pi_{1}$ be an $LHD\left(8,3\right)$ obtained from He et al. [36]:*

$$\Lambda_{1}=\left(\begin{array}{cccccccc} 0 & 0 & 0 & 0 & 0 & 0 & 0 & 0\\ 0 & 0 & 0 & 1 & 0 & 1 & 1 & 1\\ 0 & 0 & 1 & 0 & 1 & 0 & 1 & 1\\ 0 & 0 & 1 & 1 & 1 & 1 & 0 & 0\\ 0 & 1 & 0 & 0 & 1 & 1 & 0 & 1\\ 0 & 1 & 0 & 1 & 1 & 0 & 1 & 0\\ 0 & 1 & 1 & 0 & 0 & 1 & 1 & 0\\ 0 & 1 & 1 & 1 & 0 & 0 & 0 & 1 \end{array}\right),\;\;\;\;B=\left(B_{1}|B_{2}\right)=\left(\begin{array}{ccc} 0 & 0 & 0\\ 1 & 1 & 1\\ 0 & 1 & 2\\ 1 & 0 & 3 \end{array}\right)\;and\;\;$$

$$\Pi_{1}=\left(\begin{array}{ccc} 0 & 0 & 0\\ 2 & 3 & 6\\ 3 & 6 & 2\\ 1 & 5 & 4\\ 6 & 2 & 3\\ 4 & 1 & 5\\ 5 & 4 & 1\\ 7 & 7 & 7 \end{array}\right).$$

**Figure 2:**
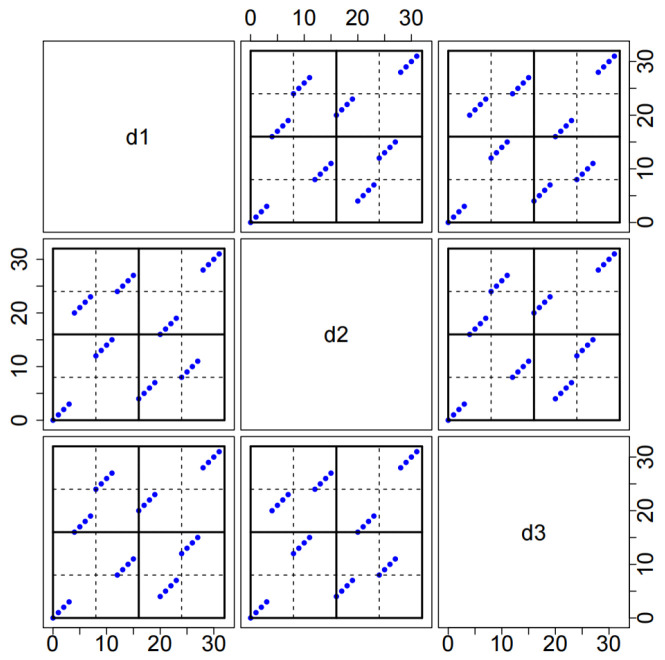
Bivariate projections among the three columns $d_{1}$, $d_{2}$, $d_{3}$ of $D_{2}$ in Example 2.

*Here, $\Pi_{1}$ qualifies as an SOA(8,3,8,3), according to He et al. [36], meaning that (i) any two distinct columns in $\Pi_{1}$ achieve stratification on 4×2 and 2×4 grids, and (ii) $\Pi_{1}$ can achieve stratification on a 2×2×2 grid in three dimensions. In Step 1, remove the first column of $\Lambda_{1}$ to obtain the matrix $\Lambda_{1}^{*}$. Construct a 32×15 matrix $D_{1}$ using $D_{1}=\left(\Lambda_{1}^{*}⊕B_{1},1_{8}⊗{\left(0,1,\ldots ,7\right)}^{T}\right)$, which is listed in Table A1 of Appendix B. For a given q=3, we can also construct $D_{2}$ from $D_{2}=J_{8\times 3}⊗B_{2}+8\Pi_{1}⊗1_{8}$, which is provided in Table A1 of Appendix B. It is easy to check that $D=(D_{1},D_{2})$ is an $MCD\left(32,2^{14}8^{1},3\right)$. The two-dimensional space-filling properties of $D_{2}$ can be seen intuitively in Figure 2, i.e., any two distinct columns in $D_{2}$ achieve stratification on 4×2 and 2×4 grids. In addition, $D_{2}$ achieves stratification on a 2×2×2 grid in three dimensions.*

Note that the subdesign of the *n* level in $D_{1}$ just has one column, which is not advisable. To increase the number of columns at level *n* in $D_{1}$, we present Algorithm 3 below, which aims to generate MCDs with $u_{2}n$ runs for $u_{2}=\lambda s^{2}$ and λ≥1. Suppose a difference scheme $D(u_{2},v_{2},n)$, denoted as $\Lambda_{2}$, and an $MCD\left(n,s^{m},p\right)$, denoted as $C=(C_{1},C_{2})$, are available, where $C_{1}$ and $C_{2}$ are an $OA\left(n,s^{m},2\right)$ and an $LHD\left(n,p\right)$, respectively.
**Algorithm 3** Construction of $MCD\left(nu_{2},s^{m}n^{v_{2}^{*}},qp\right)$ based on $MCD\left(n,s^{m},p\right)$*Step 1.* 
Let $\Lambda_{2}^{*}=\Lambda_{2}\setminus 0_{u_{2}}$, where $\Lambda_{2}\setminus 0_{u_{2}}$ is the matrix that consists of all columns in $\Lambda_{2}$ except $0_{u_{2}}$. Let $v_{2}^{*}=v_{2}-1$ if $0_{u_{2}}$ is in $\Lambda_{2}$; otherwise, let $v_{2}^{*}=v_{2}$.*Step 2.* 
Construct an $\left(nu_{2}\right)\times \left(m+v_{2}^{*}\right)$ matrix as $D_{1}=\left(1_{u_{2}}⊗C_{1},\Lambda_{2}^{*}⊕{\left(0,1,\ldots ,n-1\right)}^{T}\right)$, where the ⊕ operator is based on GF(n).*Step 3.* 
For a given q≥1, let $D_{2}=J_{u_{2}\times q}⊗C_{2}+n\Pi_{2}⊗1_{n}$, where $\Pi_{2}$ is an $LHD\left(u_{2},qp\right)$.*Step 4.* 
The resulting design $D=(D_{1},D_{2})$.

Algorithm 3 presents a method to extend the number of columns at level *n* in $D_{1}$ up to $v_{2}^{*}$. Theorem 3 summarizes the properties of $D_{1}$ and $D_{2}$ constructed in Algorithm 3.

**Theorem** **3.** *For $\Pi_{2}$ from Step 3 of Algorithm 3 and $D=(D_{1},D_{2})$ generated by Algorithm 3, the following are true:*

(i) *$D=(D_{1},D_{2})$ is an $MCD\left(nu_{2},s^{m}n^{v_{2}^{*}},qp\right)$, if $0_{u_{2}}$ is in $\Lambda_{2}$; then $v_{2}^{*}=v_{2}-1$; otherwise $v_{2}^{*}=v_{2}$;*
(ii) *$D_{2}$ and $\Pi_{2}$ achieve the same low-dimensional stratification.*

Theorem 3(ii) illustrates that the space-filling property of $D_{2}$ in Algorithm 3 is dependent on that of the LHD $\Pi_{2}$, which means that $D_{2}$ may have the desired low-dimensional space-filling property when we choose $\Pi_{2}$ with the low-dimensional space-filling property. The projection properties of $D_{2}$ in Algorithm 3 are similar to those of $D_{2}$ in Algorithm 2; therefore, $\Pi_{2}$ can also be obtained from orthogonal-array-based LHDs or strong-orthogonal-array-based LHDs.

In Algorithms 2 and 3, the $MCD\left(n,s^{m},p\right)$s may either be identical or distinct, i.e., $B=(B_{1},B_{2})$ and $C=(C_{1},C_{2})$ can be the same or different. If the number of runs in the two difference schemes $D(u_{1},v_{1},s)$ in Algorithm 2 and $D(u_{2},v_{2},n)$ in Algorithm 3 is equal, that is, $u_{1}=u_{2}$, then $\Pi_{1}$ in Algorithm 2 and $\Pi_{2}$ in Algorithm 3 can take the same LHD. Next, we construct a new space-filling asymmetrical MCD from Algorithm 3, using the same symmetrical MCD and LHD as in Algorithm 2.

**Example 3.** 
*Let $\Lambda_{2}$ be a $D\left(8,8,4\right)$ as follows:*

$$\Lambda_{2}=\left(\begin{array}{cccccccc} 0 & 0 & 0 & 0 & 0 & 0 & 0 & 0\\ 0 & 3 & 1 & 2 & 0 & 3 & 1 & 2\\ 0 & 1 & 0 & 1 & 2 & 3 & 2 & 3\\ 0 & 2 & 1 & 3 & 2 & 0 & 3 & 1\\ 0 & 0 & 2 & 2 & 1 & 1 & 3 & 3\\ 0 & 3 & 3 & 0 & 1 & 2 & 2 & 1\\ 0 & 1 & 2 & 3 & 3 & 2 & 1 & 0\\ 0 & 2 & 3 & 1 & 3 & 1 & 0 & 2 \end{array}\right).$$

*Let C=B, $C_{1}=B_{1}$, and $C_{2}=B_{2}$, where $B=(B_{1},B_{2})$ are obtained from Example 2. Here, $\Lambda_{2}^{*}$ is the matrix which deletes the first column of $\Lambda_{2}$. From Steps 1 and 2, we can obtain a 32×9 matrix $D_{1}$ using $D_{1}=\left(1_{8}⊗C_{1},\Lambda_{2}^{*}⊕{\left(0,1,\ldots ,7\right)}^{T}\right)$, which is presented in Table A2 of Appendix B. Consider the case of q=3 in Step 3. For the $LHD\left(8,3\right)$ $\Pi_{2}$, let $\Pi_{2}=\Pi_{1}$, where $\Pi_{1}$ is obtained from Example 2; then $D_{2}$ can be constructed as $D_{2}=J_{8\times 3}⊗C_{2}+8\Pi_{2}⊗1_{8}$ in Step 3, which is provided in Table A2 of Appendix B. It is easy to check that $D=(D_{1},D_{2})$ is an $MCD\left(32,2^{2}4^{7},3\right)$. The space-filling properties of $D_{2}$ are similar to those of $D_{2}$ in Example 2 and are therefore omitted here. Note that the addition operations in Step 2 are given as follows (Table 1).*

In Algorithms 2 and 3, the existence of the difference scheme is of great significance. Due to Theorem 6.6, Corollary 6.39 and Theorem 6.63 of Hedayat et al. [19], there exist three types of difference schemes, (i) $D\left(p^{w},p^{w},p^{v}\right)$; (ii) $D\left(2s^{m},2s^{m},s\right)$; and (iii) $D\left(4s^{m},4s^{m},s\right)$, where *p* is a prime, *s* is a prime power, and *w*, *v*, and *m* are positive integers, with w≥v≥1 and m≥1. Table 6.67 of Hedayat et al. [19] gives the exact maximal value of *v* for which a difference scheme $D\left(\lambda r,v,r\right)$ exists, for r=2,3,4,8,9, as listed in Table A3.

The above three algorithms can generate space-filling MCDs with $D_{1}$ being an $OA\left(2n,2^{m-1}4^{1},2\right)$, an $OA\left(nu_{1},s^{mv_{1}^{*}}n^{1},2\right)$ and $OA\left(nu_{2},s^{m}n^{v_{2}^{*}},2)\right)$, respectively. We introduce another construction for space-filling MCDs with $D_{1}$ being an $OA\left(s_{1}^{2},s_{1}^{m-1}s^{s-1}s_{2}^{1},2\right)$. Suppose an $OA\left(s^{2},s^{s+1},2\right)$, say *E*, and an $MCD\left(s_{1}^{2},s_{1}^{m},p\right)$ with $s_{1}=s^{3}$, denoted as $M=(M_{1},M_{2})$, are available, where $M_{1}$ and $M_{2}$ are an $OA\left(s_{1}^{2},s_{1}^{m},2\right)$ and an $LHD\left(s_{1}^{2},p\right)$, respectively (Algorithm 4).
**Algorithm 4** Construction of $MCD\left(s_{1}^{2},s_{1}^{m-1}s^{s-1}s_{2}^{1},p\right)$ based on $MCD\left(s_{1}^{2},s_{1}^{m},p\right)$*Step 1.* 
Let $M_{1}=\left(\Omega ,\xi \right)$, where Ω and ξ are the first (m−1) columns and the last column of $M_{1}$, respectively. Permute the rows of $M=(M_{1},M_{2})$ to obtain an MCD, denoted as $\tilde{M}=\left({\tilde{M}}_{1},{\tilde{M}}_{2}\right)$ with ${\tilde{M}}_{1}=\left(\tilde{\Omega },\tilde{\xi }\right)$, where $\tilde{\xi }={\left(0,1,\ldots ,s_{1}-1\right)}^{T}⊗1_{s_{1}}$.*Step 2.* 
For $s_{1}=s^{3}$, construct two $s_{1}\times \left(s+1\right)$ matrices $F_{0}$ and $F_{1}$ based on *E*, as $F_{0}=0_{s}⊕E$ and $F_{1}={\left(0,\ldots ,s-1\right)}^{T}⊕E\;\left(\mathrm{over}\;GF\left(s\right)\right)$, respectively.*Step 3.* 
Obtain two $s_{1}^{2}\times \left(s+1\right)$ matrices $\Phi_{0}$ and $\Phi_{1}$ by replacing the levels $0,1,\ldots ,s_{1}-1$ of the $\tilde{\xi }$ with the 1st, 2nd, …, and $s_{1}$th rows of $F_{0}$ and $F_{1}$, respectively. Let $\Phi_{0}=\left(\phi_{0}^{1},\ldots ,\phi_{0}^{s+1}\right)$ and $\Phi_{1}=\left(\phi_{1}^{1},\ldots ,\phi_{1}^{s+1}\right)$, where $\phi_{0}^{i}$ and $\phi_{1}^{i}$ are the *i*th columns of $\Phi_{0}$ and $\Phi_{1}$, respectively.*Step 4.* 
For $s_{1}=s^{3}$ and $s_{2}=s^{2}$, construct an $OA\left(s_{1}^{2},s_{1}^{m-1}s^{s-1}s_{2}^{1},2\right)$ $D_{1}$ as$$D_{1}=\left(\tilde{\Omega },\phi_{0}^{1},\ldots ,\phi_{0}^{s-1},s\phi_{0}^{s+1}+\phi_{1}^{s+1}\right).$$*Step 5.* 
Let $G=\psi ({\tilde{M}}_{2},s_{1})$. Construct three $s_{1}^{2}\times p$ matrices $H=(\phi_{1}^{1},\ldots ,\phi_{1}^{1})$, $U=(\phi_{0}^{s},\ldots ,\phi_{0}^{s})$ and $V=(\phi_{1}^{2},\ldots ,\phi_{1}^{2})$. Let $D_{2}=s^{3}G+s^{2}H+sU+V$.*Step 6.* 
The resulting design $D=(D_{1},D_{2})$.

**Theorem** **4.** *For $M=(M_{1},M_{2})$ in Algorithm 4 and $D=(D_{1},D_{2})$ generated by Algorithm 4, where $s_{1}=s^{3}$ and $s_{2}=s^{2}$, the following are true:*

(i) *$D=(D_{1},D_{2})$ is an $MCD\left(s_{1}^{2},s_{1}^{m-1}s^{s-1}s_{2}^{1},p\right)$;*
(ii) *$D_{2}$ and $M_{2}$ achieve the same low-dimensional stratification.*

Theorem 4(i) shows that the existence of an $MCD\left(s_{1}^{2},s_{1}^{m-1}s^{s-1}s_{2}^{1},p\right)$ with $s_{1}=s^{3}$ and $s_{2}=s^{2}$ is equivalent to the simultaneous existence of both an $OA\left(s^{2},s^{s+1},2\right)$ and an $MCD\left(s_{1}^{2},s_{1}^{m},p\right)$. According to He et al. [21], $m\leq s_{1}$ in an $MCD\left(s_{1}^{2},s_{1}^{m},p\right)$. For a prime *h* and a positive integer *v*, if $s=h^{v}$, there exist an $OA\left(s^{2},s^{s+1},2\right)$ and an $MCD\left(s_{1}^{2},s_{1}^{s_{1}},p\right)$.

Theorem 4(ii) tells us that the space-filling property of $D_{2}$ in Algorithm 4 is determined by that of $M_{2}$ in the initial MCD $M=(M_{1},M_{2})$. From Theorems 3.1, 3.2 and 3.20 of Hedayat et al. [19], for a prime *h* and a positive integer *v*, $s=h^{v}$, the following three OAs exist: (i) $OA\left(s^{3},s^{s^{2}+s+1},2\right)$; (ii) $OA\left(s^{3},s^{s+2},3\right)$ for h=2; (iii) and $OA\left(s^{3},s^{s+1},3\right)$ for an odd prime *h*. Thus $M=(M_{1},M_{2})$ can be obtained from Construction 1 of He et al. [21], where *M* is an $MCD\left(s_{1}^{2},s_{1}^{s_{1}},s^{2}+s+1\right)$ with $s_{1}=s^{3}$, and any two distinct columns of $M_{2}$ achieve stratification on an s×s grid. So any two distinct columns of $D_{2}$ in Theorem 4 achieve stratification on an s×s grid. According to Remark 1 of He et al. [21], for $s_{1}=s^{3}$, *M* can choose an $MCD\left(s_{1}^{2},s_{1}^{s_{1}},s+2\right)$ for *s* that is a power of 2, and an $MCD\left(s_{1}^{2},s_{1}^{s_{1}},s+1\right)$ for an odd prime power *s*, respectively; furthermore, $M_{2}$ achieves stratification in any three dimensions. Hence, the corresponding $D_{2}$ in Theorem 4 also possesses a three-dimensional space-filling property.

Based on Algorithm 4, Theorem 4 confirms that asymmetrical MCDs with attractive space-filling properties can be constructed. Next, we give an example to illustrate Algorithm 4 and Theorem 4.

**Figure 3:**
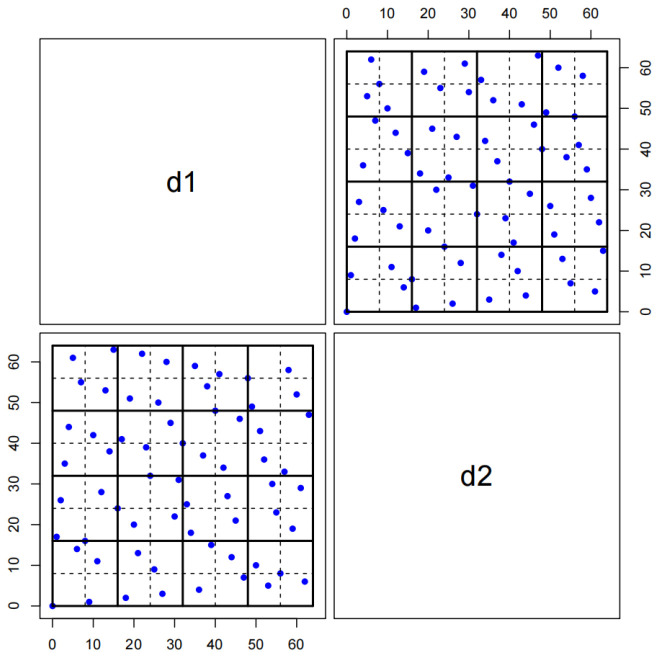
Bivariate projections between $d_{1}$ and $d_{2}$ of $D_{2}$ in Example 4.

**Example 4.** 
*Consider s=2, $s_{1}=8$, m=7, and p=2. An $OA(4,2^{3},2)$ is given as $E=\left(e_{1},e_{2},e_{3}\right)$, where $e_{1}={\left(0,0,1,1\right)}^{T}$, $e_{2}={\left(0,1,0,1\right)}^{T}$, and $e_{3}={\left(0,1,1,0\right)}^{T}$. Let $M=(M_{1},M_{2})$ be an $MCD\left(64,8^{7},2\right)$, where $M_{1}$ and $M_{2}$ are $OA\left(64,8^{7},2\right)$ and $LHD\left(64,2\right)$, respectively. In Step 1, if we permute the rows of M, we can obtain $\tilde{M}=\left({\tilde{M}}_{1},{\tilde{M}}_{2}\right)$, which is listed in Table A4 of Appendix B, where ${\tilde{M}}_{1}=\left(\tilde{\Omega },\tilde{\xi }\right)$ and $\tilde{\xi }={\left(0,1,\ldots ,7\right)}^{T}⊗1_{8}$. Obtain two 8×3 matrices $F_{0}$ and $F_{1}$ based on E using $F_{0}=0_{2}⊕E$ and $F_{1}={\left(0,1\right)}^{T}⊕E$ over GF(2). From Step 3, we can obtain two 64×3 matrices, $\Phi_{0}=\left(\phi_{0}^{1},\phi_{0}^{2},\phi_{0}^{3}\right)$ and $\Phi_{1}=\left(\phi_{1}^{1},\phi_{1}^{2},\phi_{1}^{3}\right)$. For $s_{2}=4$, we obtain $D_{1}=\left(\tilde{\Omega },\phi_{0}^{1},2\phi_{0}^{3}+\phi_{1}^{3}\right)$ in Step 4, and $G=\psi ({\tilde{M}}_{2},8)$ in Step 5. We also construct three 64×2 matrices, $H=(\phi_{1}^{1},\phi_{1}^{1})$, $U=(\phi_{0}^{s},\phi_{0}^{s})$, and $V=(\phi_{1}^{2},\phi_{1}^{2})$. The resulting designs, $D_{1}=\left(\tilde{\Omega },\phi_{0}^{1},2\phi_{0}^{3}+\phi_{1}^{3}\right)$ and $D_{2}=8G+4H+2U+V$, are listed in Table A5 of Appendix B. Next, let $d_{1}$ and $d_{2}$ be the first and second columns of $D_{2}$. It is easy to see that $d_{1}$ and $d_{2}$ achieve stratification on an 8×8 grid, as shown in Figure 3.*

## 4. Numerical Study

In this section, we validate the performance of the asymmetrical MCDs constructed by our methods in computer experiments.

Consider two $MCD\left(32,2^{2}4^{2},3\right)$s, taken from Example 3 of this paper and Example 3 of Deng et al. [18], and denote them as $\mathrm{MCD}1=(P_{1},P_{2})$ and $\mathrm{MCD}2=(Q_{1},Q_{2})$, respectively. Here, for the $MCD\left(32,2^{2}4^{7},3\right)$ $D=(D_{1},D_{2})$ listed in Table A2 of Example 3, $P_{1}$ consists of the first four columns of $D_{1}$, and $P_{2}=D_{2}$. For the $MCD\left(32,2^{8}4^{2},20\right)$ $D=(D_{1},D_{2})$ generated by Construction 3 of Deng et al. [18], as illustrated in their Example 3, $Q_{1}$ and $Q_{2}$ are taken from the last four columns of $D_{1}$ and the first three columns of $D_{2}$, respectively.

First, we compare the space-filling properties of the above MCD1 with those of the above MCD2 under the maximum projection criterion [37], the uniformity criterion [38], and the minimum mixed-moment aberration criterion [39]. For MCD1 and MCD2, Table 2 shows the values of the maximum projection criterion (“MaxProQQ”), uniformity criterion (“QQD”) and mixed-moment pattern (“MK”). Obviously, MCD1 outperforms MCD2 under the three types of criteria, which implies that MCD1 has better space-filling properties.

We next evaluate the performance of MCD1 and MCD2 in building statistical surrogate models. We conduct simulations and generate data for a computer experiment with four qualitative factors, which have two levels, two levels, four levels and four levels, respectively, and three quantitative factors. Its computer model has the following form:

$$y=f_{i}\left(x\right)\times \left(g_{j}\left(x\right)+h_{k}\left(x\right)\right)+t_{l}\left(x\right),x=\left(x_{1},x_{2},x_{3}\right),$$

where *i*, *j*, *k*, and *l* are the levels for the qualitative factors, i,j=1,2, k,l=1,2,3,4; $0\leq x_{1},x_{2},x_{3}\leq 1$ are the values of quantitative factors; and the functions $f_{i}$, $g_{j}$, $h_{k}$, and $t_{l}$ are as shown below:

$f_{1}\left(x\right)=x_{1}+x_{2}^{2}+x_{3}^{3}$, $f_{2}\left(x\right)=x_{1}^{2}+x_{2}+x_{3}^{3}$,

$g_{1}\left(x\right)=cos\left(x_{1}\right)+cos\left(2x_{2}\right)+cos\left(3x_{3}\right)$, $g_{2}\left(x\right)=cos\left(3x_{1}\right)+cos\left(2x_{2}\right)+cos\left(x_{3}\right)$,

$h_{1}\left(x\right)=sin\left(x_{1}\right)+sin\left(2x_{2}\right)+sin\left(3x_{3}\right)$, $h_{2}\left(x\right)=sin\left(3x_{1}\right)+sin\left(2x_{2}\right)+sin\left(x_{3}\right)$, $h_{3}\left(x\right)=sin\left(2x_{1}\right)+sin\left(x_{2}\right)+sin\left(3x_{3}\right)$, $h_{4}\left(x\right)=sin\left(x_{1}\right)+sin\left(3x_{2}\right)+sin\left(2x_{3}\right)$,

$t_{1}\left(x\right)=e^{x_{1}}+e^{x_{2}}+e^{x_{3}}$, $t_{2}\left(x\right)=e^{2x_{1}}+e^{x_{2}}+e^{x_{3}}$, $t_{3}\left(x\right)=e^{x_{1}}+e^{2x_{2}}+e^{x_{3}}$, $t_{4}\left(x\right)=e^{x_{1}}+e^{x_{2}}+e^{2x_{3}}$.

We adopt the easy-to-interpret Gaussian process model first proposed by Xiao et al. [40] to fit the data corresponding to MCD1 and MCD2, and use the root-mean-square prediction error (RMSE) to measure the prediction performance. The RMSE is as follows:

$$\mathrm{RMSE}=\sqrt{\frac{1}{n_{t}}\sum_{i=1}^{n_{t}}{\left(y\left(z_{i}^{*}\right)-\hat{y}\left(z_{i}^{*}\right)\right)}^{2}},$$

where $z_{i}^{*}$ is a test point on the test point set, $i=1,2,\ldots ,n_{t}$, and $y(z_{i}^{*})$ and $\hat{y}\left(z_{i}^{*}\right)$ are the true and predicted responses of the input $z_{i}^{*}$.

**Figure 4:**
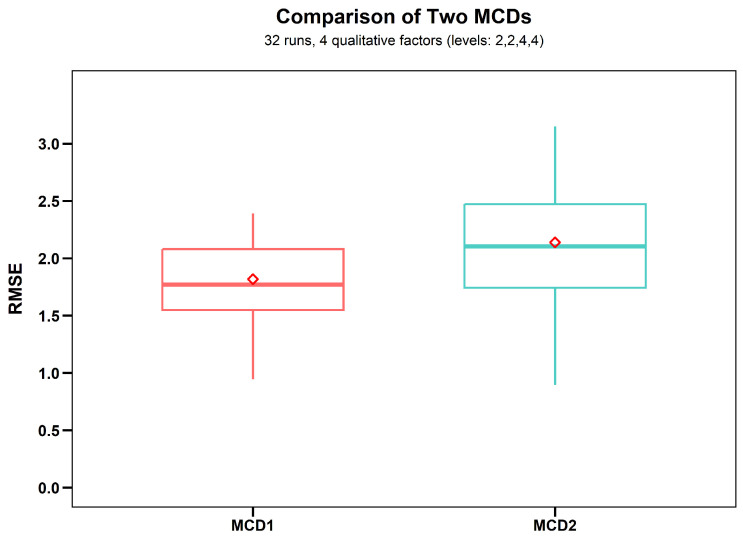
The prediction results of MCD1 and MCD2, where the red square represent the mean of RMSE.

According to the RMSE in Figure 4, MCD1 certainly outperforms MCD2 in building statistical surrogate models.

## 5. Concluding Remarks

Although Construction 3 of Deng et al. [18] constructs asymmetrical MCDs, it does not discuss the space-filling property of the designs for quantitative factors in MCDs. Using simple and easily implementable Algorithms 1, 2, 3 and 4, the $MCD\left(2n,2^{m-1}4^{1},p\right)$, $MCD\left(nu_{1},s^{mv_{1}^{*}}n^{1},qp\right)$, $MCD\left(nu_{2},s^{m}n^{v_{2}^{*}},qp\right)$, and $MCD\left(s_{1}^{2},s_{1}^{m-1}s^{s-1}s_{2}^{1},p\right)$ with $s_{1}=s^{3}$ and $s_{2}=s^{2}$ are obtained, respectively. Based on symmetrical MCDs with good low-dimensional projection properties in designs for quantitative factors, Algorithms 1 and 4 construct a series of asymmetrical MCDs, which inherit these low-dimensional space-filling properties. Algorithms 2 and 3 make use of the symmetrical MCDs and the space-filling LHDs to generate asymmetrical MCDs with desirable space-filling properties. Compared with Zhou et al. [24], the designs obtained by Algorithms 1–3 are very flexible in terms of their run sizes: (i) if an asymmetrical MCD is constructed by Algorithm 1, the run size of such a design is a multiple of 8; (ii) when constructing the asymmetrical MCDs using Algorithms 2 and 3, their run sizes must be of the form $\lambda s^{2}$ for some integer λ.

The number of qualitative factors in an MCD determines its range of applications, so it is important to determine the upper bound of the number of qualitative factors. For the $MCD\left(2n,2^{m-1}4^{1},p\right)$ constructed by Algorithm 1, the $MCD\left(nu_{1},s^{mv_{1}^{*}}n^{1},qp\right)$ generated by Algorithm 2, the $MCD\left(nu_{2},s^{m}n^{v_{2}^{*}},qp\right)$ obtained by Algorithm 3, and the $MCD\left(s_{1}^{2},s_{1}^{m-1}s^{s-1}s_{2}^{1},p\right)$ obtained by Algorithm 4, on the one hand, since $v_{i}^{*}=v_{i}-1$ or $v_{i}^{*}=v_{i}$ for i=1,2, the maximum value of $v_{i}^{*}$ can be obtained from Table A3. On the other hand, according to Equation (3.2) of [21], it is known that m≤n/2 in $MCD\left(2n,2^{m-1}4^{1},p\right)$, m≤n/s in $MCD\left(nu_{1},s^{mv_{1}^{*}}n^{1},qp\right)$, m≤n/s in $MCD\left(nu_{2},s^{m}n^{v_{2}^{*}},qp\right)$, and $m\leq s_{1}$ in $MCD\left(s_{1}^{2},s_{1}^{m-1}s^{s-1}s_{2}^{1},p\right)$.

Given a small initial MCD $D^{\left(0\right)}=\left(D_{1}^{\left(0\right)},D_{2}^{\left(0\right)}\right)$ with $D_{1}^{\left(0\right)}$ and $D_{2}^{\left(0\right)}$ being an OA and an LHD(n,p), respectively, from Construction 3 of Deng et al. [18], the large MCD $D=\left(D_{1},D_{2}\right)$ is found to be $D_{1}=D\left(1\right)⊕D_{1}^{\left(0\right)}$ and $D_{2}=C⊗D_{2}^{\left(0\right)}+nH⊗1_{n}$, where D(1) is a difference scheme, $C=\left(c_{ij}\right)$ is a u×f matrix with $c_{ij}=1$, and *H* is an $LHD\left(u,pf\right)$. Obviously, the space-filling property of $D_{2}$ depends on *H*, since $\psi (D_{2},n)=H$ from Equation (1). So we can choose a MCD from this paper as the initial small asymmetrical MCD and select a space-filling *H* to construct a series of large space-filling asymmetrical MCDs based on Construction 3 of Deng et al. [18].

As we know, orthogonality is extremely important for fitting polynomial models. A natural direction for future work would be to construct good asymmetrical MCDs with orthogonality.

## Figures and Tables

**Table 1:** Addition table for GF(4) in Example 3.

+	0	1	2	3
0	0	1	2	3
1	1	0	3	2
2	2	3	0	1
3	3	2	1	0

The operation symbol “+” represents addition in the Galois field GF(4).

**Table 2:** The values of different designs in the numerical study.

	MaxProQQ	QQD	MK = (MK_1_, MK_2_, MK_3_, MK_4_)
MCD1	10.7032	0.0542	(0.3876, 0.2623, 0.2359, 0.2564)
MCD2	12.8260	0.0968	(0.4358, 0.3792, 0.4488, 0.6300)

## Data Availability

The original contributions presented in this study are included in the article. Further inquiries can be directed to the corresponding authors.

## Appendix A. Proofs

To prove Theorem 1, Lemma A1 is given, which is derived from Remark 3 of Li et al. [41].

**Lemma A1.** 

*Suppose $A^{*}$ is an $OA\left(n,2^{m},2\right)$ based on GF(2); then*

$$A=\left(\begin{array}{cc} 0_{n} & A^{*}\\ 1_{n} & A^{*}+1 \end{array}\right)$$

*is an $OA\left(2n,2^{m+1},3\right)$, where $A^{*}+1$ is the matrix obtained by adding 1 (mod 2) to all the entries of $A^{*}$.*

**Proof of Theorem 1.** (i) For i,k=1,2,…,m and i≠k, $\left(a_{11}^{0},a_{11}^{i},a_{11}^{k}\right)$ is an $OA(2n,2^{3},3)$, which follows from Lemma A1. Thus, $D_{1}$ is an $OA\left(2n,2^{m-1}4^{1},2\right)$. For j=1,2,…,p, let $a_{2}^{j}$ and $d_{2}^{j}$ be the *j*th columns of $A_{2}$ and $D_{2}$, respectively. Clearly, $a_{2}^{j}$ and $d_{2}^{j}$ are an LHD(n,1) and LHD(2n,1), respectively. From the construction of $a_{11}^{i}$ and $\psi \left(d_{2}^{j},2\right)=1_{2}⊗a_{2}^{j}$, we can determine that $\left(a_{11}^{1},a_{11}^{2},\ldots ,a_{11}^{m-1},\psi \left(d_{2}^{j},2\right)\right)$ is an $OA\left(2n,2^{m-1}n^{1},2\right)$. Moreover, $\left(a_{11}^{0},a_{11}^{i},\psi \left(d_{2}^{j},4\right)\right)$ being an $OA\left(2n,2^{2}{\left(n/2\right)}^{1},3\right)$ follows, since $a_{11}^{i}={\left(0,1\right)}^{T}⊕a_{1}^{i}$ and $\psi \left(d_{2}^{j},4\right)=1_{2}⊗\psi \left(a_{2}^{j},2\right)$, and $\left(a_{1}^{i},\psi \left(a_{2}^{j},2\right)\right)$ is an $OA\left(n,2^{1}{\left(n/2\right)}^{1},2\right)$. So $\left(2a_{11}^{m-1}+a_{11}^{0},\psi \left(d_{2}^{j},4\right)\right)$ is an $OA\left(2n,4^{1}{\left(n/2\right)}^{1},2\right)$. Now Part (i) can be proven from Lemma 1. (ii) According to the construction of $D_{2}$, it is easy to see that $D_{2}$ and $A_{2}$ achieve the same low-dimensional stratification. □

**Proof of Theorem 2.** (i) $D_{1}$ being an $OA\left(nu_{1},s^{mv_{1}^{*}}n^{1},2\right)$ follows from Theorem 9.15 of Section 9.3 of Hedayat et al. [19]. From Construction 3 of Deng et al. [18], we can find that $D_{2}$ is an $LHD\left(nu_{1},qp\right)$, and $\left(\Lambda_{1}^{*}⊕B_{1},D_{2}\right)$ is an $MCD\left(nu_{1},s^{mv_{1}^{*}},qp\right)$. Let $\pi_{i}$ be the *i*th column of $\Pi_{1}⊗1_{n}$, i=1,2,…,pq. Since $\psi \left(D_{2},n\right)=\Pi_{1}⊗1_{n}$, and $\left(1_{u_{1}}⊗{\left(0,1,\cdots n-1\right)}^{T},\pi_{i}\right)$ for i=1,2,…,pq is an $OA\left(nu_{1},n^{1}u_{1}^{1},2\right)$, then $\left(1_{u_{1}}⊗{\left(0,1,\cdots n-1\right)}^{T},D_{2}\right)$ is an $MCD\left(nu_{1},n^{1},qp\right)$ according to Lemma 1. Then (i) can be obtained straightforwardly. (ii) From Algorithm 2, it is easy to see that $\psi \left(D_{2},n\right)=\Pi_{1}⊗1_{n}$. Now Part (ii) can be proven. □

**Proof of Theorem 3.** Let $u_{2}=\beta n$ and n=ηs, where β and η are positive integers. $\Lambda_{2}^{*}⊕{\left(0,1,\cdots ,n-1\right)}^{T}$ being an $OA\left(nu_{2},n^{v_{2}^{*}},2\right)$ follows from Lemma 6.27 of Section 9.3 of Hedayat et al. [19]. It is clear that $1_{u_{2}}⊗C_{1}$ is an $OA\left(nu_{2},s^{m},2\right)$. For i=1,2,…,m, let $c_{1}^{i}$ and $1_{u_{2}}⊗c_{1}^{i}$ be the *i*th columns of $C_{1}$ and $1_{u_{2}}⊗C_{1}$, respectively. Let $\lambda_{k}$ be the *k*th column of $\Lambda_{2}^{*}⊕{\left(0,1,\cdots ,n-1\right)}^{T}$, $k=1,2,\ldots ,v_{2}^{*}$. For any α∈GF(n), $\left(c_{1}^{i},\alpha ⊕{\left(0,1,\cdots n-1\right)}^{T}\right)$ then occurs β times in the rows of $\left(1_{u_{2}}⊗c_{1}^{i},\lambda_{k}\right)$, i=1,2,…,m, $k=1,2,\ldots ,v_{2}^{*}$, since $\Lambda_{2}^{*}$ is a difference scheme $D(u_{2},v_{2},n)$. Thus, for i=1,2,…,m, $k=1,2,\ldots ,v_{2}^{*}$, $\left(1_{u_{2}}⊗c_{1}^{i},\lambda_{k}\right)$ is an $OA\left(nu_{2},s^{1}n^{1},2\right)$. So, $D_{1}$ is an $OA\left(nu_{2},s^{m}n^{v_{2}^{*}},2\right)$. Similarly to the proof of Theorem 2, $D_{2}$ is an $LHD\left(nu_{2},qp\right)$. Let $d_{i}$ and $\pi_{i}$ be the *i*th columns of $D_{2}$ and $\Pi_{2}$, respectively, i=1,2,…,pq. Let $c_{2}^{i}$ is the *i*th column of $C_{2}$, where i=1,2,…,p. For i=1,2,…,pq, $\psi \left(d_{i},n\right)=\pi_{i}⊗1_{n}$, and there exists *k* such that $\psi \left(d_{i},s\right)=1_{u_{2}}⊗\psi \left(c_{2}^{k},s\right)+\eta \pi_{i}⊗1_{n}$, k=1,2,…,p. It is easy to see that for $k=1,2,\ldots ,v_{2}^{*}$, i=1,2,…,pq, $\left(\lambda_{k},\psi \left(d_{i},n\right)\right)$ is an $OA\left(nu_{2},n^{1}u_{2}^{1},2\right)$. For j=1,2,…,m, k=1,2,…,p and i=1,2,…,pq, $\left(1_{u_{2}}⊗c_{1}^{j},1_{u_{2}}⊗\psi \left(c_{2}^{k},s\right),\pi_{i}⊗1_{n}\right)$ is an $OA\left(nu_{2},s^{1}\eta^{1}u_{2}^{1},3\right)$, since $\left(c_{1}^{j},\psi \left(c_{2}^{k},s\right)\right)$ is an $OA\left(n,s^{1}\eta^{1},2\right)$. Thus $\left(1_{u_{2}}⊗c_{1}^{j},\psi \left(d_{i},s\right)\right)$ is an $OA\left(nu_{2},s^{1}{\left(\eta u_{2}\right)}^{1},2\right)$ for j=1,2,…,m and i=1,2,…,pq. Thus, Theorem 3(i) is true according to Lemma 1, since $u_{2}=\beta n$ and n=ηs. (ii) Similarly to the proof of Theorem 2(ii), it is straightforward to see that (ii) is true. □

To prove Theorem 4, the following two Lemmas are given. Following the symbols in Algorithm 4, let $F_{0}=\left(f_{0}^{1},\ldots ,f_{0}^{s+1}\right)$ and $F_{1}=\left(f_{1}^{1},\ldots ,f_{1}^{s+1}\right)$, where $f_{0}^{i}$ and $f_{1}^{i}$ are the *i*th columns of $F_{0}$ and $F_{1}$, respectively.

Lemma A2 is from Wang et al. [42].

**Lemma A2.** 

*Suppose $f_{0}^{i}$, $f_{0}^{j}$, $f_{0}^{k}$, $f_{1}^{i}$, $f_{1}^{j}$ and $f_{1}^{k}$ for i,j,k=1,2,…,s+1, i≠j are as defined above; then the arrays $\left(f_{0}^{i},f_{0}^{j},f_{1}^{k}\right)$ and $\left(f_{1}^{i},f_{1}^{j},f_{0}^{k}\right)$ are $OA\left(s_{1},s^{3},3\right)$s with $s_{1}=s^{3}$.*

Following the symbols $G=\psi ({\tilde{M}}_{2},s_{1})$, $\phi_{0}^{i}$ and $\phi_{1}^{i}$, i=1,2,…,s+1 in Algorithm 4, let $G=(g_{1},\ldots ,g_{p})$, where $g_{i}$ is the *i*th column of *G*.

**Lemma A3.** 

*For u=1,2,…,p and i=1,2,…,s+1, suppose $g_{u}$, $\phi_{0}^{i}$, and $\phi_{1}^{i}$ are as defined above; then for i≠j the arrays $\left(g_{u},\phi_{0}^{i},\phi_{0}^{j},\phi_{1}^{k}\right)$ and $\left(g_{u},\phi_{1}^{i},\phi_{1}^{j},\phi_{0}^{k}\right)$ are $OA\left(s_{1}^{2},s_{1}s^{3},4\right)$s with $s_{1}=s^{3}$.*

**Proof of Lemma A3.** For u=1,2,…,p, the $g_{u}$ defined above, and ξ in Step 1 of Algorithm 4, $\left(g_{u},\xi \right)$ is an $OA\left(s_{1}^{2},s_{1}^{2},2\right)$ according to Proposition 1 of He et al. [21]. Note that after row permutation, $\left(g_{u},\tilde{\xi }\right)=\left(1_{s_{1}}⊗{\left(0,1,\ldots ,s_{1}-1\right)}^{T},{\left(0,1,\ldots ,s_{1}-1\right)}^{T}⊗1_{s_{1}}\right)$. It is clear that the arrays $\left(g_{u},\phi_{0}^{i},\phi_{0}^{j},\phi_{1}^{k}\right)$ and $\left(g_{u},\phi_{1}^{i},\phi_{1}^{j},\phi_{0}^{k}\right)$ are $OA\left(s_{1}^{2},s_{1}s^{3},4\right)$s with $s_{1}=s^{3}$, since the arrays $\left(f_{0}^{i},f_{0}^{j},f_{1}^{k}\right)$ and $\left(f_{1}^{i},f_{1}^{j},f_{0}^{k}\right)$ are $OA\left(s_{1},s^{3},3\right)$s with $s_{1}=s^{3}$ according to Lemma A2. □

**Proof of Theorem 4.** (i) It is clear that $D_{1}$ being an $OA\left(s_{1}^{2},s_{1}^{m-1}s^{s-1}s_{2}^{1},2\right)$ follows from the expansive replacement method of Section 9.3 of Hedayat et al. [19]. Let $g_{i}$ and $d_{i}$ be the *i*th columns of *G* and $D_{2}$, respectively, where i=1,2,…,p. For i=1,2,…,p, $d_{i}=s^{3}g_{i}+s^{2}\phi_{1}^{1}+s\phi_{0}^{s}+\phi_{1}^{2}$. For i=1,2,…,p, $d_{i}$ being an $LHD\left(s_{1}^{2},1\right)$ follows from Lemma A3. Thus, $D_{2}$ is an $LHD\left(s_{1}^{2},p\right)$. From Algorithm 4, it is obvious that $\left(\tilde{\Omega },\psi \left(d_{i},s_{1}\right)\right)$ is an $OA\left(s_{1}^{2},s_{1}^{m},2\right)$ for i=1,2,…,p, based on $\left(M_{1},M_{2}\right)$ being an $MCD\left(s_{1}^{2},s_{1}^{m},p\right)$ and $G=\psi ({\tilde{M}}_{2},s_{1})$. According to Lemmas A2 and A3, for i=1,2,…,p, $s_{1}=s^{3}$, $s_{2}=s^{2}$, $\left(\phi_{0}^{1},\ldots ,\phi_{0}^{s-1},\psi \left(d_{i},s\right)\right)$, and $\left(s\phi_{0}^{s+1}+\phi_{1}^{s+1},\psi \left(d_{i},s_{2}\right)\right)$, *i* is an $OA\left(s_{1}^{2},s^{s-1}{\left(s^{5}\right)}^{1},2\right)$ and an $OA\left(s_{1}^{2},s_{2}{\left(s^{4}\right)}^{1},2\right)$, respectively. From Lemma 1, $D=(D_{1},D_{2})$ is an $MCD\left(s_{1}^{2},s_{1}^{m-1}s^{s-1}s_{2}^{1},p\right)$ with $s_{1}=s^{3}$ and $s_{2}=s^{2}$. (ii) From Construction 1, it is easy to see that $\psi ({\tilde{M}}_{2},s_{1})=G$ and $\psi (D_{2},s_{1})=G$. Then (ii) can be obtained straightforwardly. □

## Appendix B. Tables

**Table A1:** $MCD\left(32,2^{14}4^{1},3\right)$$D=(D_{1},D_{2})$ in Example 2.

Run	$D_{1}$															$D_{2}$		
1	0	0	0	0	0	0	0	0	0	0	0	0	0	0	0	0	0	0
2	1	1	1	1	1	1	1	1	1	1	1	1	1	1	1	1	1	1
3	0	1	0	1	0	1	0	1	0	1	0	1	0	1	2	2	2	2
4	1	0	1	0	1	0	1	0	1	0	1	0	1	0	3	3	3	3
5	0	0	0	0	1	1	0	0	1	1	1	1	1	1	0	8	12	24
6	1	1	1	1	0	0	1	1	0	0	0	0	0	0	1	9	13	25
7	0	1	0	1	1	0	0	1	1	0	1	0	1	0	2	10	14	26
8	1	0	1	0	0	1	1	0	0	1	0	1	0	1	3	11	15	27
9	0	0	1	1	0	0	1	1	0	0	1	1	1	1	0	12	24	8
10	1	1	0	0	1	1	0	0	1	1	0	0	0	0	1	13	25	9
11	0	1	1	0	0	1	1	0	0	1	1	0	1	0	2	14	26	10
12	1	0	0	1	1	0	0	1	1	0	0	1	0	1	3	15	27	11
13	0	0	1	1	1	1	1	1	1	1	0	0	0	0	0	4	20	16
14	1	1	0	0	0	0	0	0	0	0	1	1	1	1	1	5	21	17
15	0	1	1	0	1	0	1	0	1	0	0	1	0	1	2	6	22	18
16	1	0	0	1	0	1	0	1	0	1	1	0	1	0	3	7	23	19
17	1	1	0	0	0	0	1	1	1	1	0	0	1	1	0	24	8	12
18	0	0	1	1	1	1	0	0	0	0	1	1	0	0	1	25	9	13
19	1	0	0	1	0	1	1	0	1	0	0	1	1	0	2	26	10	14
20	0	1	1	0	1	0	0	1	0	1	1	0	0	1	3	27	11	15
21	1	1	0	0	1	1	1	1	0	0	1	1	0	0	0	16	4	20
22	0	0	1	1	0	0	0	0	1	1	0	0	1	1	1	17	5	21
23	1	0	0	1	1	0	1	0	0	1	1	0	0	1	2	18	6	22
24	0	1	1	0	0	1	0	1	1	0	0	1	1	0	3	19	7	23
25	1	1	1	1	0	0	0	0	1	1	1	1	0	0	0	20	16	4
26	0	0	0	0	1	1	1	1	0	0	0	0	1	1	1	21	17	5
27	1	0	1	0	0	1	0	1	1	0	1	0	0	1	2	22	18	6
28	0	1	0	1	1	0	1	0	0	1	0	1	1	0	3	23	19	7
29	1	1	1	1	1	1	0	0	0	0	0	0	1	1	0	28	28	28
30	0	0	0	0	0	0	1	1	1	1	1	1	0	0	1	29	29	29
31	1	0	1	0	1	0	0	1	0	1	0	1	1	0	2	30	30	30
32	0	1	0	1	0	1	1	0	1	0	1	0	0	1	3	31	31	31

**Table A2:** $MCD\left(32,2^{2}4^{7},3\right)$$D=(D_{1},D_{2})$ in Example 3.

Run	$D_{1}$									$D_{2}$			Run	$D_{1}$									$D_{2}$		
1	0	0	0	0	0	0	0	0	0	0	0	0	17	0	0	0	2	2	1	1	3	3	24	8	12
2	1	1	1	1	1	1	1	1	1	1	1	1	18	1	1	1	3	3	0	0	2	2	25	9	13
3	0	1	2	2	2	2	2	2	2	2	2	2	19	0	1	2	0	0	3	3	1	1	26	10	14
4	1	0	3	3	3	3	3	3	3	3	3	3	20	1	0	3	1	1	2	2	0	0	27	11	15
5	0	0	3	1	2	0	3	1	2	8	12	24	21	0	0	3	3	0	1	2	2	1	16	4	20
6	1	1	2	0	3	1	2	0	3	9	13	25	22	1	1	2	2	1	0	3	3	0	17	5	21
7	0	1	1	3	0	2	1	3	0	10	14	26	23	0	1	1	1	2	3	0	0	3	18	6	22
8	1	0	0	2	1	3	0	2	1	11	15	27	24	1	0	0	0	3	2	1	1	2	19	7	23
9	0	0	1	0	1	2	3	2	3	12	24	8	25	0	0	1	2	3	3	2	1	0	20	16	4
10	1	1	0	1	0	3	2	3	2	13	25	9	26	1	1	0	3	2	2	3	0	1	21	17	5
11	0	1	3	2	3	0	1	0	1	14	26	10	27	0	1	3	0	1	1	0	3	2	22	18	6
12	1	0	2	3	2	1	0	1	0	15	27	11	28	1	0	2	1	0	0	1	2	3	23	19	7
13	0	0	2	1	3	2	0	3	1	4	20	16	29	0	0	2	3	1	3	1	0	2	28	28	28
14	1	1	3	0	2	3	1	2	0	5	21	17	30	1	1	3	2	0	2	0	1	3	29	29	29
15	0	1	0	3	1	0	2	1	3	6	22	18	31	0	1	0	1	3	1	3	2	0	30	30	30
16	1	0	1	2	0	1	3	0	2	7	23	19	32	1	0	1	0	2	0	2	3	1	31	31	31

**Table A3:** The maximum *v* such that a $D\left(\lambda r,v,r\right)$ exists, r=2,3,4,8,9.

λ	$D\left(2\lambda ,v,2\right)$	$D\left(3\lambda ,v,3\right)$	$D\left(4\lambda ,v,4\right)$	$D\left(8\lambda ,v,8\right)$	$D\left(9\lambda ,v,9\right)$
	v	v	v	v	v
1	2	3	4	8	9
2	4	6	8	16	18
3	2	9	12	-	27
4	8	12	16	32	36
6	12	18	-	-	54
7	2	-	-	56	-
8	16	24	32	64	72
9	2	27	36	-	81

**Table A4:** $\tilde{M}=\left({\tilde{M}}_{1},{\tilde{M}}_{2}\right)$ in Example 4.

	${\tilde{M}}_{1}$										${\tilde{M}}_{1}$								
Run	$\tilde{\Omega }$						$\tilde{\xi }$	${\tilde{M}}_{2}$		Run	$\tilde{\Omega }$						$\tilde{\xi }$	${\tilde{M}}_{2}$	
1	0	0	0	0	0	0	0	0	0	33	0	4	4	4	4	4	4	36	36
2	1	1	2	3	4	5	0	48	56	34	1	2	1	6	0	7	4	28	44
3	2	2	3	4	5	6	0	56	8	35	2	1	6	0	7	3	4	44	20
4	3	3	4	5	6	7	0	8	16	36	3	6	0	7	3	5	4	20	12
5	4	4	5	6	7	1	0	16	24	37	4	0	7	3	5	2	4	12	52
6	5	5	6	7	1	2	0	24	32	38	5	7	3	5	2	1	4	52	4
7	6	6	7	1	2	3	0	32	40	39	6	3	5	2	1	6	4	4	60
8	7	7	1	2	3	4	0	40	48	40	7	5	2	1	6	0	4	60	28
9	0	1	1	1	1	1	1	9	9	41	0	5	5	5	5	5	5	45	45
10	1	0	4	7	2	6	1	41	25	42	1	6	3	2	7	0	5	13	37
11	2	4	7	2	6	5	1	25	1	43	2	3	2	7	0	1	5	37	53
12	3	7	2	6	5	3	1	1	33	44	3	2	7	0	1	4	5	53	29
13	4	2	6	5	3	0	1	33	57	45	4	7	0	1	4	6	5	29	21
14	5	6	5	3	0	4	1	57	17	46	5	0	1	4	6	3	5	21	61
15	6	5	3	0	4	7	1	17	49	47	6	1	4	6	3	2	5	61	5
16	7	3	0	4	7	2	1	49	41	48	7	4	6	3	2	7	5	5	13
17	0	2	2	2	2	2	2	18	18	49	0	6	6	6	6	6	6	54	54
18	1	4	0	5	1	3	2	58	50	50	1	5	7	4	3	1	6	6	22
19	2	0	5	1	3	7	2	50	34	51	2	7	4	3	1	0	6	22	46
20	3	5	1	3	7	6	2	34	2	52	3	4	3	1	0	2	6	46	62
21	4	1	3	7	6	4	2	2	42	53	4	3	1	0	2	5	6	62	38
22	5	3	7	6	4	0	2	42	10	54	5	1	0	2	5	7	6	38	30
23	6	7	6	4	0	5	2	10	26	55	6	0	2	5	7	4	6	30	14
24	7	6	4	0	5	1	2	26	58	56	7	2	5	7	4	3	6	14	6
25	0	3	3	3	3	3	3	27	27	57	0	7	7	7	7	7	7	63	63
26	1	7	5	0	6	2	3	35	11	58	1	3	6	1	5	4	7	23	7
27	2	5	0	6	2	4	3	11	59	59	2	6	1	5	4	2	7	7	31
28	3	0	6	2	4	1	3	59	43	60	3	1	5	4	2	0	7	31	55
29	4	6	2	4	1	7	3	43	3	61	4	5	4	2	0	3	7	55	15
30	5	2	4	1	7	5	3	3	51	62	5	4	2	0	3	6	7	15	47
31	6	4	1	7	5	0	3	51	19	63	6	2	0	3	6	1	7	47	39
32	7	1	7	5	0	6	3	19	35	64	7	0	3	6	1	5	7	39	23

**Table A5:** $D=\left(D_{1},D_{2}\right)$ in Example 4, $h=2\phi_{0}^{3}+\phi_{1}^{3}$.

	$D_{1}$											$D_{1}$									
Run	$\tilde{\Omega }$						$\phi_{0}^{1}$	h	$D_{2}$		Run	$\tilde{\Omega }$						$\phi_{0}^{1}$	h	$D_{2}$	
1	0	0	0	0	0	0	0	0	0	0	33	0	4	4	4	4	4	0	1	37	37
2	1	1	2	3	4	5	0	0	48	56	34	1	2	1	6	0	7	0	1	29	45
3	2	2	3	4	5	6	0	0	56	8	35	2	1	6	0	7	3	0	1	45	21
4	3	3	4	5	6	7	0	0	8	16	36	3	6	0	7	3	5	0	1	21	13
5	4	4	5	6	7	1	0	0	16	24	37	4	0	7	3	5	2	0	1	13	53
6	5	5	6	7	1	2	0	0	24	32	38	5	7	3	5	2	1	0	1	53	5
7	6	6	7	1	2	3	0	0	32	40	39	6	3	5	2	1	6	0	1	5	61
8	7	7	1	2	3	4	0	0	40	48	40	7	5	2	1	6	0	0	1	61	29
9	0	1	1	1	1	1	0	3	11	11	41	0	5	5	5	5	5	0	2	46	46
10	1	0	4	7	2	6	0	3	43	27	42	1	6	3	2	7	0	0	2	14	38
11	2	4	7	2	6	5	0	3	27	3	43	2	3	2	7	0	1	0	2	38	54
12	3	7	2	6	5	3	0	3	3	35	44	3	2	7	0	1	4	0	2	54	30
13	4	2	6	5	3	0	0	3	35	59	45	4	7	0	1	4	6	0	2	30	22
14	5	6	5	3	0	4	0	3	59	19	46	5	0	1	4	6	3	0	2	22	62
15	6	5	3	0	4	7	0	3	19	51	47	6	1	4	6	3	2	0	2	62	6
16	7	3	0	4	7	2	0	3	51	43	48	7	4	6	3	2	7	0	2	6	14
17	0	2	2	2	2	2	1	3	20	20	49	0	6	6	6	6	6	1	2	49	49
18	1	4	0	5	1	3	1	3	60	52	50	1	5	7	4	3	1	1	2	1	17
19	2	0	5	1	3	7	1	3	52	36	51	2	7	4	3	1	0	1	2	17	41
20	3	5	1	3	7	6	1	3	36	4	52	3	4	3	1	0	2	1	2	41	57
21	4	1	3	7	6	4	1	3	4	44	53	4	3	1	0	2	5	1	2	57	33
22	5	3	7	6	4	0	1	3	44	12	54	5	1	0	2	5	7	1	2	33	25
23	6	7	6	4	0	5	1	3	12	28	55	6	0	2	5	7	4	1	2	25	9
24	7	6	4	0	5	1	1	3	28	60	56	7	2	5	7	4	3	1	2	9	1
25	0	3	3	3	3	3	1	0	31	31	57	0	7	7	7	7	7	1	1	58	58
26	1	7	5	0	6	2	1	0	39	15	58	1	3	6	1	5	4	1	1	18	2
27	2	5	0	6	2	4	1	0	15	63	59	2	6	1	5	4	2	1	1	2	26
28	3	0	6	2	4	1	1	0	63	47	60	3	1	5	4	2	0	1	1	26	50
29	4	6	2	4	1	7	1	0	47	7	61	4	5	4	2	0	3	1	1	50	10
30	5	2	4	1	7	5	1	0	7	55	62	5	4	2	0	3	6	1	1	10	42
31	6	4	1	7	5	0	1	0	55	23	63	6	2	0	3	6	1	1	1	42	34
32	7	1	7	5	0	6	1	0	23	39	64	7	0	3	6	1	5	1	1	34	18
